# Peer effects of depression between left-behind and non-left-behind children: quasi-experimental evidence from rural China

**DOI:** 10.1186/s13034-023-00602-1

**Published:** 2023-06-12

**Authors:** Li Huang, Sizhe Zhang, Biyu Bian, Mi Zhou, Zinan Bi

**Affiliations:** College of Economics and Management, Shenyang Agricultural University, Shenyang, 110866 Liaoning China

**Keywords:** Peer contagion, Rural children, Friendship, Teacher–student relationships

## Abstract

**Purpose:**

The aim of the study is to investigate the interactive influence of depression on left-behind (LB) and non-left-behind (NLB) children from the perspective of peer effects. The roles of teachers, parents, and friends are also explored.

**Methods:**

Data on 1817 children, 1817 parents, and 55 teachers were obtained from a field survey in December 2021. All students in the sample were randomly assigned to classrooms. A peer effect model and OLS methods were used to estimate the peer influence of depression. Robustness tests were conducted by randomly removing schools from the sample.

**Results:**

Depression was contagious among different groups of rural children, and the peer effect of the NLB children’s depression played a dominant role. Both LB and NLB children were more affected by their NLB classmates’ depression. LB children were not significantly affected by depression in other LB children. This conclusion remains robust after robustness testing. In addition, heterogeneity analysis showed that outgoing and cheerful teachers, effective parent–child communication and high-quality friendship all alleviated peer influence on depression.

**Conclusions:**

LB children have more severe depression than NLB children, but LB children are more affected by depression in their NLB peers. Policymakers should train teachers to engage in positive communication with students to improve mental health in children. In addition, this article recommends that children move and live with their parents when family conditions permit.

## Introduction

Children and adolescents are more likely to experience psychological problems because of their unstable self-esteem, limited life experience, and lower resistance to setbacks [62]. According to data released by the China Health Commission in 2018, about 30 million Chinese children under the age of 17 suffer from various emotional disorders, learning problems and behavioral problems caused by psychological stress. A 2020 meta-analysis estimated that 17.2% of Chinese children ages 6–15 reported depressive symptoms [58]. If left untreated, childhood depression may increase the potential for mental health difficulties and suicidal behavior in adulthood [17, 26].

Rural areas of China are underdeveloped and are some of the hardest hit by mental illness, with psychological problems frequently reported in children [30]. A previous study found that children living in rural China are statistically significantly more likely to be depressed than those living in urban areas (22.7% vs. 13.5%) [63, 64]. Furthermore, there are many left-behind children living in rural China. According to data from China's seventh population census, there were over 61.02 million left-behind children in rural China, accounting for 21.88% of all children in the country. A long-term lack of parental love and care makes rural left-behind children more vulnerable to mental illnesses like anxiety and depression [36, 51, 63, 64].

Susceptibility to peer effects increases throughout childhood and peaks during adolescence (Steinberg and Monahan [54]). Most prior research in this area has focused on the role of peer effects in amplifying problematic behavior [14, 52]. However, emotional distress as a peer effect in children and adolescents is an important topic of research, especially given that incidences of depression are increasing (Costello et al. [32]), and depression has high continuity into adulthood (Gutman and Sameroff [28], Rudolph et al. [47]). Psychology studies have shown that emotions can be contagious [18, 31, 53]. Stevens and Prinstein [55] found that a adolescent having depressive symptoms predicted future depressive symptoms in their friends and that negative thinking styles were also influenced by peers.

This paper is interested in the peer effect of depression between two groups of rural children, namely left-behind (LB) and non-left-behind (NLB) children. The term “LB” children in this paper refers to rural-registered minors whose parents are migrant workers who do not live with their children consistently. To be specific, this paper uses the peer effect model and random assignment data from field investigations to analyze the interactive influence of LB and NLB children’s depression scores on others’ scores within these groups.

### Identification of peer effects

The key challenge in identifying peer effects is the reflection problem posed by Manski [38]. The implication of this problem is that when researchers try to infer whether the average behavior of a group affects the behavior of the individual, the causal relationship between the influence of students on their peers cannot be distinguished since individual behavior and peer behavior are determined simultaneously.

According to Manski [38], the reflection problem is divided into three parts: the endogenous effect, the exogenous effect and the correlated effect. The endogenous effect means that individuals may change their own behavior as others in their group do. The exogenous effect refers to an individual’s tendency to behave in some way that varies with the exogenous characteristics of the group (such as the family background of peers). The correlated effects include two aspects: one is the correlated group factor, which describes children’s tendency to behave similarly because they have similar individual characteristics (which is often referred to as the sample self-selection problem in empirical studies). The other is common environmental factors, that is, groups with similar environments, such as a similar school environment, exhibit similar behaviors.

Manski [38] pointed out that in a linear model, the expectation of endogenous effects and that of exogenous effects would produce complete collinearity, and it was therefore difficult to distinguish them even assuming that correlated effects are completely excluded. Therefore, the peer effects explored in this paper include endogenous effects and exogenous effects. To exclude the common environmental factors of the correlated effect, as much information as possible (including the characteristics of schools, classes and teachers) can be added into the econometric model as control variables [7, 25]. Removing student choice behavior can help combat the correlated group factor of the correlated effects (for example, [65]). Fortunately, the children in the sample used for this study are from schools where students are randomly assigned to classrooms, which eliminates the problem of sample self-selection.

### Peer effects in different groups of children

Existing literature on peer effects between different groups of children has focused on immigrant students and native students, or students of different races, and most of the literature focuses on impacts on cognitive ability or school achievement [2, 9, 12, 20, 25, 29, 33, 49]. However, there is little literature examining peer effects of depression among different groups of children. Some studies have found that immigrant students have no or positive effects on the performance of domestic students [12, 49], and other studies have also found that immigrant students have a negative impact on domestic students [25, 33]. At the same time, such studies have found that the size of the peer effect is related to the proportion of different types of students in the class. Entorf and Lauk [16] found that the peer effect of migrant students on both native and migrant students is significantly lower than that of native students on these two groups. Billings et al. [4] found that both white and minority students score lower on high school exams when they are assigned to schools with more minority students [4]. Frattini and Meschi [19] also explore the effect that immigrant peers have on native student achievement and found that the peer effect of having immigrant classmates is only observable in classes with a high immigrant concentration (in the top 20%).

The first hypothesis (Hypothesis 1) in this study states that depression has a significant peer effect between LB children and NLB children. In the sample, the proportion of LB children in the class was only 27.8% on average. Hence, the following hypothesis (Hypothesis 2) is put forward: the peer effect of NLB children plays a dominant role within the class.

### Heterogeneity of peer effects

Studies have shown that family and school are important factors leading to depression in children and adolescents. Meanwhile, peer relationships are also closely related to childhood depression [1, 42, 62]. First, teachers’ personalities can have deep, persistent and latent impact on children, and teachers are important figures in school mental health education. Extraversion relates to social interaction and affects how a person acts in a social context [21, 24, 35]. Studies have shown that having an extraverted teacher is associated with positive interpersonal contact between students, which may directly or indirectly affect the psychological development of students [11, 35]. Second, parent–child communication is usually defined as the verbal and nonverbal interaction between parents and children within a family system [59]. In general, higher levels of parent–child communication were related to lower levels of depression [37]. Third, depression is also associated with problematic peer relationships [5, 6]. However, friendships can serve as a buffer for peer group depression [3, 41]. Previous studies have shown that regardless of the level of group acceptance, children without friends were lonelier than children who had at least one mutual friendship. Additionally, friendships are generally beneficial for psychological development, except when friends have adjustment problems or friendships are of low quality [13, 50].

Based on the above literature, it is also hypothesized that teacher personalities, parent–child communication and peer relationships moderate the depression contagion between LB children and NLB children (Hypothesis 3).

Previous studies have analyzed the effects of depression on peer effect from the perspective of rural left-behind children and non-left-behind children [60, 61], (Rui et al. 34), and found that the incidence of depression in rural left-behind children and non-left-behind children is higher than average, but little literature has analyzed the effects between them.

Unlike these previous studies, this paper analyzes the interactive influence of LB and NLB children’s depressive moods on one another. This paper is the first to directly study the peer effect of depression between these two groups of children, which supplements the existing literature on peer effects and mental health of left-behind children. The conclusions of this study are valuable for the governments of population inflow areas and can help them formulate policies and promote child development. Furthermore, the results provide useful guidelines for drawing up relevant policies in other developing countries that also have a large number of domestic migrants.

## Methods

### Data collection procedures

The data observed in this study are from a research survey conducted in December 2021 in Xinzhuang Town, Puyang City, Henan Province. Henan is a traditionally agricultural province and has the largest population in central China. All primary and secondary school students in the area were surveyed. The enrollment rate of the school-age population, the enrollment rate from primary school to junior high school, and the coverage rate of 9-year compulsory education in Xinzhuang are all 100%. Younger children lack the reading comprehension skills necessary to complete the survey, so only children from grades 4 to 9 were investigated. The questionnaires include a rich set of questions about the children’s development, family background, and class and school environment. They were pretested before fieldwork. To improve response quality, children filled out the questionnaires with trained survey coordinators and teachers present to help them with any questions or difficulties.

A total of 55 classes in 16 primary and secondary schools surveyed 1817 rural children. A total of 1817 guardians (the parents of the children or other relatives who were able to describe the real situation of everyone in the whole family) and 55 teachers (the head teacher of each class) participated.

### Measures

#### Left-behind children

This study defines left-behind children as children who have one or more of their parents leave them to work for 6 or more consecutive months [15, 60, 61].

#### Depression

Depression scores were measured using the CES-D scale. The CES-D scale includes 20 items and is scored on a Likert scale, with four possible answers corresponding to how often the respondents experienced a given emotion within the past week: “rarely or none of the time (less than 1 day)”, “some or a little of the time (1–2 days)”, “occasionally or a moderate amount of time (3–4 days)”, and “most or all of the time (5–7 days)”. Possible scores range from 0 to 60, and a score of 17 or higher is indicative of depression [45, 63, 64]. The Chinese version of the CES-D has been shown to be appropriate for use in China, and its reliability and validity has been tested among Chinese populations in prior research [27]. The Cronbach’s alpha coefficient for the current sample was 0.84.

#### Demographics

Child demographic questions included the child’s gender (1 = male, 0 = female), age, whether the child was an only child (1 = yes, the child is an only child, 0 = no, the child has siblings), whether the child was a boarder (1 = yes, the student lives in school dormitories, 0 = no, the child does not live at school), the child’s health status (1 = very healthy or healthy, 0 = very unhealthy, unhealthy or neutral) and the child’s degree of mental development. Degree of mental development was evaluated by presenting the students with the following two situations: “When I have different views from my classmates, I can think of ways to prevent conflicts” (Mental Development Degree 1) and “It is normal for different people to use different methods to do the same thing” (Mental Development Degree 2), where 1 = completely agree or mostly agree and 0 = completely disagree, mostly disagree or unsure. In addition, because this paper only focuses on the emotional contagion spread offline within the class, time spent playing online games is also controlled (to exclude possible emotional contagion between the children via internet communication).

Parent and Family questions included guardian education level (1 = junior high school or above, 0 = other), guardian occupation (1 = farmer, 0 = other) and family economic status. Family economic status was obtained by taking the logarithm of the family’s annual income as reported on the Parent Questionnaire.

The characteristic variables related to the correlated effects, including class size, as well as the teacher’s tenure, gender (1 = male, 0 = female), education (1 = master’s degree or above; 0 = other), and professional title (1 = senior professional title; 0 = other). Class size was a continuous variable measured in numbers and ranging from 10 to 63. Length of head teacher's teaching career was a continuous variable measured in years and taking the logarithm.

#### Teacher’s extraversion

This article measures teacher extraversion through three questions: (i) “I like to talk to people,” (ii) “I am outgoing and social,” and (iii) “I am conservative.” The third question is processed in reverse. Teachers are asked to rate the extent to which they agree with the statements on a scale from 1 (strongly disagree) to 5 (strongly agree). The scores of the three questions are averaged, and then extraversion = 1 if the average score is greater than or equal to 4, and 0 if otherwise. The internal reliability of the scale in the sample was good (⍺ = 0.80).

#### Active caretaker communication

In this article, active communication between caretaker and child was measured with the caretakers’ responses to the following question: "Do you actively discuss your child's concerns or worries with him or her?" Active communication = 1 if the caretakers selected the option “Often,” and 0 if they answered “Never” or “Occasionally.”

#### Friendship quality and quantity

To measure friendship quality, we constructed an index using two survey items from the student questionnaire: (i) “I think most of my friends trust me,” and (ii) “My friends make me feel good about my ideas.” Meanwhile, the friendship quantity index is measured by the following item: “I think I have a lot of friends.” Students were asked to rate the extent to which they agree with the statements on a scale from 1 (strongly disagree) to 5 (strongly agree).

### Econometric model

In this paper, the sample was divided into left-behind and non-left behind children. The average depression scores for the LB and NLB children were then examined for their influence on their peers’ scores (within the same class). The specific model settings are as follows:

1 $$Depression_{i}^{l} = \beta_{l}^{l} *\frac{1}{{N_{l} - 1}}\mathop \sum \limits_{j\, = 1}^{{N_{l} \, - 1}} Depression_{j} + \beta_{c}^{l} *\frac{1}{{N_{c} }}\mathop \sum \limits_{k\, = 1}^{{N_{c} }} Depression_{k} + \beta_{1} X_{i} + \beta_{2} Correlated_{i} + D_{s} + u_{i}$$

2 $$Depression_{i}^{c} = \beta_{c}^{c} *\frac{1}{{N_{c} - 1}}\mathop \sum \limits_{p\, = 1}^{{N_{c} - 1}} Depression_{p} + \beta_{l}^{c} *\frac{1}{{N_{l} }}\mathop \sum \limits_{q\, = 1}^{{N_{l} }} Depression_{q} + \beta_{1} X_{i} + \beta_{2} Correlated_{i} + D_{s} + u_{i}$$

$Depression_{i}^{l}$ represents the depression score of a given LB child i, and $Depression_{i}^{c}$ represents the depression score of a given NLB child i.

In model (1), $\frac{1}{{N_{l} - 1}}\mathop \sum \limits_{j = 1}^{{N_{l} - 1}} Depression_{j}$ represents the average depression score of other LB children in the class (excluding LB child i), and $\frac{1}{{N_{c} }}\mathop \sum \limits_{k = 1}^{{N_{c} }} Depression_{k}$ represents the average depression score of the NLB children in the class. The regression coefficients $\beta_{l}^{l}$ and $\beta_{c}^{l}$ respectively indicate the degree of change in LB child i’s depression score when the average depression scores of other LB or NLB children in the class increase by one point, that is, the peer effect.

Depression is not only influenced by peers, but also by a child’s personal, family and school characteristics. ${X}_{i}$ represents the control variables, including gender, age, only-child status, boarding-student status, health status, degree of mental development, time spent playing online games, parental education level, parental occupation and family economic status. The control variable ${Correlated}_{i}$ represents the characteristic variables related to the correlated effects, including class size, as well as the head teacher's teaching career, gender, education, and professional title. $D_{s}$ is the school–grade fixed effect. $u_{i}$ is the error term. Equation (2) shares a similar model setting with Eq. (1), but takes the NLB child as its research object. We cluster standard errors at the class level, accounting for correlation in outcomes for students in the same class.

### Data analysis

First, descriptive analysis was performed. Descriptive statistics (means and standard deviations for continuous variables and percentages for categorical variables) were used to describe study variables. Second, a balance testing method from Gong et al. [23] was used to further verify the reliability of the "random assignment" of the sample. Third, an OLS model was used to estimate the interactive influence of left-behind and non-left behind children’s depression on fellow LB and NLB classmates. As random class assignment is conducted within a school and students may nonrandomly select their school, school fixed effects were included in the regressions to control for all school-level factors in the cross-sectional data that may influence students’ school selection decisions. Standard errors were also clustered at the class level, accounting for correlation in outcomes for students in the same class, and significance set at the p < 0.5 level. Finally, the robustness test drew from Gong et al. [22] by randomly deleting schools from the sample to observe whether there were significant changes in the regression results. All analyses were conducted using Stata 16.1 software [43].

The definitions and descriptive statistics for the variables are shown in Table A1. On average, rural children had a depression score of 15.81. The LB children’s average depression score (16.126) is higher than that of the NLB children (15.695).

### Balancing test

Although we determined through interviews with the principal of each school and the head teacher of each class that all students in our sample were randomly assigned to classes, we also drew on the balance testing method used in Gong et al. [23] to further verify the reliability of the random assignment of our sample. If class assignment is indeed random, students with different proportions of LB peers should be similar in terms of their observed characteristics. We regressed the student’s predetermined characteristics—gender, age, health status, family economic status, parental education, only-child status, boarding-student status, class size, and the career duration, gender, education, professional title and age of the child’s teacher—on the proportion of LB children in his or her classroom.

Table 1 presents the results of the balancing test. Column (1) reports the unconditional estimates, and Column (3) reports the conditional estimates with school fixed effects. The results show that the proportion of LB children has no significant relationship with most of the student, family and teacher characteristics examined, suggesting that these characteristics are well-balanced across classrooms with different proportions of LB children. This supports the assertion that the students in our sample were randomly assigned. This finding is further verified in the robustness test section of this paper.

**Table 1:** Balancing test for predetermined characteristics

Variables	Unconditional test		Conditional test	
	Coefficient (1)	SE (2)	Coefficient (3)	SE (4)
Gender	0.102	(0.125)	0.119	(0.102)
Age	− 1.485	(1.266)	− 3.149***	(0.822)
Only child	− 0.011	(0.032)	− 0.067	(0.052)
Boarder	0.248	(0.271)	− 0.032	(0.057)
Health status	0.101	(0.090)	0.134	(0.088)
Family economic status	0.889**	(0.317)	0.547	(0.342)
Parent’s education	0.152	(0.100)	0.290*	(0.111)
Class size	− 22.057	(13.599)	− 3.810	(5.008)
Teacher's career duration	− 1.718	(0.909)	− 2.068	(1.299)
Teacher's gender	0.234	(0.387)	0.220	(0.395)
Teacher's education	− 0.096	(0.503)	− 0.300	(0.570)
Teacher's professional title	− 0.176	(0.334)	− 0.277	(0.470)
Teacher's age	− 8.189	(7.730)	− 10.412	(9.273)

Each cell represents a separate regression that regresses the corresponding predetermined characteristic above on the proportion of LB children in the class. Conditional estimates are obtained from regressions that include school fixed effects. Standard errors are clustered at the class level and reported in parentheses. Significance: *** p < 0.001, ** p < 0.01, * p < 0.05

## Main results

### Peer effects on depression scores: LB and NLB children

This paper studies the interactive influence of left-behind and non-left behind children’s depression on fellow LB and NLB classmates. The main regression results are shown in Table 2. Columns (1) and (3) report the results of adding all control variables, and columns (2) and (4) further add the school-grade fixed effect on the basis of columns (1) and (3). The results show that the significant peer effect coefficients were all positive, i.e., the influence of depression between LB and NLB children in the class worked in the same direction. This is in line with the conclusions in emotional contagion literature, that is, that emotions can be contagious [44, 55]. Additionally, NLB children dominate the peer effect between LB and NLB children, in other words, both groups are more affected by their NLB classmates’ depression, and less affected by their LB classmates’ depression. Furthermore, the LB children’s average depression scores have no significant influence on their fellow LB classmates’ scores.

**Table 2:** Estimates of Peer effects on depression scores

Variables	LB children		NLB children	
	(1)	(2)	(3)	(4)
LB classmates’ depression scores	− 0.152	− 0.426	0.167***	0.269**
	(0.157)	(0.244)	(0.046)	(0.082)
NLB classmates’ depression scores	0.622***	0.816***	0.574***	0.394**
	(0.133)	(0.177)	(0.077)	(0.116)
Student and parent controls	Yes	Yes	Yes	Yes
Correlated effects controls	Yes	Yes	Yes	Yes
School-grade fixed effects	No	Yes	No	Yes
Observations	483	483	1334	1334
R-squared	0.130	0.220	0.142	0.188

Student and parent controls include the child’s gender, age, only-child status, boarding-student status, adjusted self-rated health, mental development, time spent playing online games, guardian’s education, parent’s occupation and family economic status. Correlated effect controls include class size, head teacher’s career duration, gender, education, professional job title and age. Standard errors are clustered at the class level and reported in parentheses. The weight of the inclusion is 1/sample. Significance: *** p < 0.001, ** p < 0.01, * p < 0.05

Specifically, for the LB students, the average depression scores of their NLB classmates have a significant positive impact on their depression scores, with an influence coefficient of 0.816 [as per the regression results in column (2)]. In other words, for every one-point increase in the NLB classmates’ average depression scores, the examined LB child’s depression score increases by 0.816 points (p < 0.001). In contrast, the average depression scores of other LB children in the class have no significant impact on the given LB child’s score, indicating that, on the whole, the LB children’s depression is mainly affected by their NLB peers. For NLB children, the average depression scores of other NLB children in the class have a significant positive impact (= 0.394, p < 0.01), as shown in the regression results in column (4). The average depression scores of the LB classmates also have a significant positive impact on the NLB children’s depression scores (= 0.269, p < 0.01). The NLB children’s depression scores are, on the whole, more affected by their NLB peers.

### Robustness testing

**Fig. 1:**
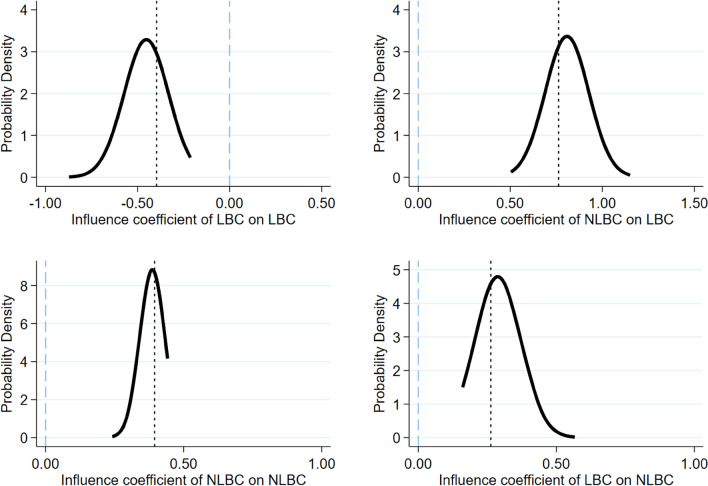
Density function diagrams of peer effect coefficients

Robustness testing was performed to further verify that non-random class assignment is not occurring within the sample. We randomly removed schools from the sample and observed whether there was a significant change in the regression results. If the students are in fact randomly assigned to classrooms, then the regression results using the random subsample should not deviate much from the benchmark regression results. To ensure the size of the sample, two schools were randomly deleted each time, and 500 regressions were performed. Figure 1 shows the density function of the peer effect coefficients for LB and NLB children in the random subsample regression. The black dotted line is the coefficient value of the benchmark regression. After random deletion, the coefficients of the samples are concentrated near the value of the benchmark regression coefficient, indicating that the benchmark regression results are not biased due to the inclusion of non-random data.

### Heterogeneity of peer effects

Panel A in Table 3 shows the results of the interactive influence of LB and NLB children’s depression in classes with extraverted vs. introverted teachers. The results show that teacher extraversion can significantly moderate student depression. The coefficients in columns (1) and (3) are significantly negative for both LB and NLB children.

**Table 3:** Results of sub-sample estimates

Variables	LB children		NLB children	
	(1)	(2)	(3)	(4)
	Extraversion	Non-extraversion	Extraversion	Non-extraversion
Panel A: Teacher personality				
LB classmates’ depression scores	− 5.526***	− 0.958*	− 9.835**	0.442***
	(1.146)	(0.380)	(2.629)	(0.112)
NLB classmates’ depression scores	− 9.438***	1.310***	− 14.286**	− 0.165
	(1.789)	(0.346)	(3.997)	(0.235)
Observations	100	383	236	1,098
R-squared	0.808	0.306	0.741	0.232

	LB children		NLB children	
	(1)	(2)	(3)	(4)
	Active	Not active	Active	Not active
Panel B: Active parent–child communication				
LB classmates’ depression scores	− 0.166	− 0.657*	0.430*	0.339**
	(0.228)	(0.299)	(0.207)	(0.122)
NLB classmates’ depression scores	0.858***	0.719**	0.207*	0.354**
	(0.173)	(0.223)	(0.082)	(0.103)
Observations	223	260	636	698
R-squared	0.314	0.315	0.193	0.223

	LB children		NLB children	
	(1)	(2)	(3)	(4)
	High quantity	Low quantity	High quantity	Low quantity
Panel C: Friendship				
LB classmates’ depression scores	− 0.180	− 0.802	0.143*	0.518***
	(0.196)	(0.433)	(0.059)	(0.141)
NLB classmates’ depression scores	0.623***	1.165**	0.534***	0.133
	(0.144)	(0.403)	(0.148)	(0.150)
Observations	276	207	784	550
R-squared	0.227	0.329	0.182	0.199

	LB children		NLB children	
	(1)	(2)	(3)	(4)
	High quality	Low quality	High quality	Low quality
LB classmates’ depression scores	− 0.100	− 0.402	0.081	0.486**
	(0.270)	(0.282)	(0.071)	(0.170)
NLB classmates’ depression scores	0.459*	0.814**	0.490	0.110
	(0.218)	(0.245)	(0.270)	(0.153)
Observations	224	259	687	647
R-squared	0.237	0.322	0.158	0.219
Student and parent controls	Yes	Yes	Yes	Yes
Correlated effects controls	Yes	Yes	Yes	Yes
School-grade fixed effects	Yes	Yes	Yes	Yes

Student and parent controls include the child’s gender, age, only-child status, boarding-student status, adjusted self-rated health, mental development, time spent playing online games, guardian’s education, parent’s occupation and family economic status. Correlated effect controls include class size, head teacher’s career duration, gender, education, professional job title and age. Standard errors are clustered at the class level and reported in parentheses. The weight of the inclusion is 1/sample. Significance: *** p < 0.001, ** p < 0.01, * p < 0.05

Panel B in Table 3 reports the results of the interactive influence of LB and NLB children’s depression for children with active caregiver–child communication vs. those without. We found that for NLB children, active communication from caregivers could reduce the impact of other children’s depression. For LB children, however, we found the opposite. Active caregiver-child communication in these children increased the influence of their NLB classmates’ depression on their own. This may be due to the fact that caregivers of LB children are usually grandparents or other people besides their parents, who tend to have less intimate relationships with them than their parents do. Therefore, such communication is less effective and can even have the reverse effect.

As shown in Table 3, Panel C, both the quantity and quality of LB children’s friendships moderated the effect of their NLB classmates’ depression scores. For NLB children, however, the quantity and quality of their friendships only moderated the effects of their LB classmates’ depression scores on their own. We found that depression in NLB children was contagious only for those with many friends, and had no significant effect on NLB children with few.

## Discussion

This paper used random class assignment data to study the peer effect of depression between LB and NLB children in rural China. We found that depression was contagious among different groups of rural children, and that the NLB children’s scores played a dominant role. Both LB and NLB children were more affected by the NLB children's depression, while NLB children were less affected by the LB children's depression, and LB children were not significantly affected by their LB classmates’ depression. The basic conclusions of this paper on the peer effect of the two types of children's depression were consistent with the results of previous studies on the cognitive ability of native and immigrant students [4, 16, 19].

The results of our heterogeneity analysis showed that teachers, parents and friends all played a significant role in the process of depressive emotional contagion among rural children. Firstly, upon entering the school system, children transition from spending the majority of their time in a home environment with parents to spending it in a classroom environment with teachers. Thus, the classroom constitutes an increasingly important environment during adolescence. Interaction between teachers and students is particularly important in all aspects of the classroom environment [56]. Several studies have revealed that having a supportive teacher can foster good behavior [8, 10] and helps students’ mental health [39, 56]. Our study confirms these findings. The results show that a teacher’s extraversion can significantly moderate his/her students’ depression. For example, when students have negative emotional reactions to test results or conflicts with classmates, teachers with high levels of extraversion typically demonstrate understanding and caring, and will actively communicate with and pacify students.

Secondly, it has been confirmed in the literature that when children feel valued, supported, and loved during their interactions with parents, they internalize feelings of positive self‐esteem [40]. A positive view of self has been found to be the primary contributor to children's psychological well‐being. In our study, positive communication between NLB children and their parents alleviated the contagion effect of peer depression. For LB children on the other hand, the positive communication that their parents provide cannot be replaced by their present caregivers. The absence of their parents thus adversely affects the children's growth and mental health. For this reason, active and positive communication from the LB children’s caregivers had a very limited effect on the contagion of peer depression for these children.

Finally, friendship is the most important peer relationship as children grow and move into adolescence. For many adolescents, relations with friends are critical interpersonal bridges that move them toward psychological growth and social maturity [46, 48]. When adolescents encounter problems, the significant physical and cognitive development in adolescence makes them more likely to turn to friends with whom they share experiences. High-quality friendships not only help children overcome adverse life events, but also have lasting positive effects on their mental health [57]. It is worth noting that the effect of the number of friends on the contagion of depression is complex, as it mainly depends on the average psychological status of one’s friends. If a child’s friends have lower depression scores, then having more friends is a protective factor for that child’s mental health. However, if many of the child’s friends have poor mental health themselves, the number of friends will not buffer the negative effects of these friends’ depression scores on the child’s mental health.

Our findings make several contributions to the literature and have some practical implications. First, our findings provide useful information for school administrators who seek to reduce student depressive symptoms. Schools should actively guide children and adolescents to maintain healthy and positive psychological states. Children should be encouraged to participate in lively and diverse campus sociocultural activities, and schools should strive for an atmosphere conducive to the healthy growth and well-rounded development of their students. Schools can provide timely, effective and high-quality mental health education and services for primary and secondary school students through individual, group, telephone, or online consultations, or by providing exciting group activities for classes, mental health education, and psychological behavior training. In addition, policy makers should give more attention to the importance of training teachers to engage in positive communication with students in order to improve adolescent mental health. Second, these results also provide insight for policymakers regarding how to improve the mental health of left-behind children. For example, schools and communities can help fill gaps in parental supervision and family education that LB children experience, making up for the negative impact of the parent–child separation effect on the LB children. This article recommends that children be encouraged to move with their parents when family conditions permit. The government should work to solve the problem of schooling for migrant children, guaranteeing migrant children’s right to go to school near their parents and obtain an education of equal quality.

This study faces some limitations. First, the findings rely on survey data that may be prone to measurement and reporting errors. Another limitation is that the sample is restricted to a single province in China. Nevertheless, the sample is representative and well-suited to test the theoretical predictions laid out in this paper and represents an interesting case in a low-income setting. Further research using more representative data from different countries is needed to test if the reported relationships hold in other settings and to further improve understanding of peer effects on children’s depression in developing countries. In addition, as traditional linear models cannot effectively distinguish endogenous effects from exogenous effects, future research should consider using spatial econometric models to identify the endogenous effects.

## Data Availability

Data are available from the authors upon reasonable request

## Appendix

See Table 4.

**Table 4:** Descriptive statistics of control variables used in the multivariate analysis

Variables	Definition	Full sample		LB child		NLB child		H0: [3] = [5]
		Mean	SD	Mean	SD	Mean	SD	Difference
		[1]	[2]	[3]	[4]	[5]	[6]	[7]
Depression scores	CES-D scores	15.810	9.267	16.126	9.338	15.695	9.242	− 0.431
Gender	1 = male; 0 = female	0.495	0.500	0.555	0.497	0.474	0.499	− 0.081**
Age	Continuous variable (year)	11.331	1.496	11.317	1.580	11.337	1.465	0.020
Only child	1 = yes; 0 = no	0.048	0.214	0.064	0.245	0.042	0.201	− 0.022
Boarder	1 = yes; 0 = no	0.165	0.371	0.199	0.399	0.153	0.360	− 0.046*
Adjusted self-rated health	1 = very healthy/healthy; 0 = very unhealthy/unhealthy/neutral	0.649	0.477	0.665	0.473	0.644	0.479	− 0.021
Mental development degree 1	"When I have different views from my classmates, I can think of ways to prevent conflicts", where 1 = completely agree/mostly agree and 0 = completely disagree/mostly disagree/unsure	0.510	0.500	0.520	0.500	0.507	0.500	− 0.013
Mental development degree 2	"It is normal for different people to use different methods to do the same thing", where 1 = completely agree/mostly agree and 0 = completely disagree/mostly disagree/unsure	0.685	0.465	0.681	0.467	0.686	0.464	0.005
Parent’s education	1 = junior high school or above; 0 = others	0.125	0.331	0.143	0.350	0.119	0.324	− 0.024
Parent’s occupation	1 = farmer; 0 = others	0.613	0.487	0.466	0.499	0.666	0.472	0.200***
Family economic status	Family’s annual income (taking the logarithm)	10.764	0.535	10.920	0.501	10.707	0.536	− 0.213***
Teacher's career duration	Continuous variable (taking the logarithm)	2.103	1.115	2.009	1.145	2.137	1.103	0.128*
Teacher's gender	1 = male; 0 = female	0.200	0.400	0.199	0.399	0.201	0.401	0.002
Teacher's education	1 = Master degree and above; 0 = others	0.020	0.139	0.025	0.156	0.018	0.133	− 0.007
Teacher's professional title	1 = Senior professional title; 0 = others	0.082	0.274	0.079	0.270	0.083	0.276	0.005
Teacher's age	Continuous variable (year)	35.656	8.387	35.369	8.480	35.760	8.354	0.392
Class size	Number	38.008	12.972	35.930	11.585	38.761	13.364	2.831***
Online game intensity 1	Average daily time spent playing online games: do not play online games	0.155	0.362	0.108	0.310	0.172	0.377	0.064***
Online game intensity 2	Average daily time spent playing online games: less than 30 min	0.389	0.488	0.356	0.479	0.401	0.490	0.045
Online game intensity 3	Average daily time spent playing online games: 30 min-1 h	0.347	0.476	0.383	0.487	0.334	0.472	− 0.049
Online game intensity 4	Average daily time spent playing online games: more than 1 h	0.109	0.312	0.153	0.361	0.093	0.290	− 0.060***
Observations	Number	1817		483		1334		

Significance: *** p < 0.001, ** p < 0.01, * p < 0.05
